# Safety and reliability of Radio Frequency Identification Devices in Magnetic Resonance Imaging and Computed Tomography

**DOI:** 10.1186/1754-9493-4-2

**Published:** 2010-02-02

**Authors:** Thomas Steffen, Roger Luechinger, Simon Wildermuth, Christian Kern, Christian Fretz, Jochen Lange, Franc H Hetzer

**Affiliations:** 1 Department of Surgery, Hospital of the Canton of St Gallen (KSSG), CH-9007 St Gallen, Switzerland; 2 Institute for Biomedical Engineering, University and ETH Zurich, CH-8091 Zurich, Switzerland; 3 InfoMedis AG, CH-6055 Alpnach, Switzerland; 4 Department of Radiology, Hospital of the Canton of St Gallen (KSSG), CH-9007 St Gallen, Switzerland

## Abstract

**Background:**

Radio Frequency Identification (RFID) devices are becoming more and more essential for patient safety in hospitals. The purpose of this study was to determine patient safety, data reliability and signal loss wearing on skin RFID devices during magnetic resonance imaging (MRI) and computed tomography (CT) scanning.

**Methods:**

Sixty RFID tags of the type I-Code SLI, 13.56 MHz, ISO 18000-3.1 were tested: Thirty type 1, an RFID tag with a 76 × 45 mm aluminum-etched antenna and 30 type 2, a tag with a 31 × 14 mm copper-etched antenna. The signal loss, material movement and heat tests were performed in a 1.5 T and a 3 T MR system. For data integrity, the tags were tested additionally during CT scanning. Standardized function tests were performed with all transponders before and after all imaging studies.

**Results:**

There was no memory loss or data alteration in the RFID tags after MRI and CT scanning. Concerning heating (a maximum of 3.6°C) and device movement (below 1 N/kg) no relevant influence was found. Concerning signal loss (artifacts 2 - 4 mm), interpretability of MR images was impaired when superficial structures such as skin, subcutaneous tissues or tendons were assessed.

**Conclusions:**

Patients wearing RFID wristbands are safe in 1.5 T and 3 T MR scanners using normal operation mode for RF-field. The findings are specific to the RFID tags that underwent testing.

## Background

Incorrect patient identification is a common clinical problem and confusions may have severe consequences, especially in surgery. Due to the increasing dispersion of electronic data processing applications in hospitals and the lack of patient identification in such systems, the correct determination of identity becomes more important than ever in hospitals. The radio frequency identification (RFID) technology with tags (transponders) in wristbands and medical equipment may close this gap and increase patient safety.

An RFID tag is a microchip attached to an antenna and usually fashioned in such a way that it can be fixed to an object. The tag picks up signals from and sends signals to a reader. Most RFID tags work in a passive mode without an own source of energy and transmit signals only on demand from a reader. RFID-systems work at different frequency bands: the low-frequency (LF) systems typically work at ranges from 125 - 134.2 kHz, high-frequency (HF) systems work at 13.56 MHz and transponders at ultra high frequencies (UHF) work from 868 - 950 MHz or even up to 2.4 GHz.

The first RFID applications for hospitals have recently been tested successfully and implemented for instance to track blood bottles, drugs, dispensers and other small containers or documents. RFID-systems for transfusion safety have been successfully developed and tested [1-4]. Correct patient identification is still the most important key element in these processes and should be integrated in any advanced hospital RFID system beyond simple logistic applications.

Initial attempts in the field of human RFID tagging have been described for disaster victim identification. The most preferred method there was an embedded RFID tag in dental prostheses [5,6].

Another implanted system for humans, whereby the tag is placed under the skin, has been developed and was approved by the U.S. Food and Drug Administration (FDA) in 2004. This system called VeriChip™ was recommended for example for patient identification [5,7,8]. This implantable device has been successfully tested as MR conditional up to 7 T in cylindrical systems. No adverse effects for patients were found in this small test series of only two implanted devices [8]. However, the instruction of the device recommends a continuous monitoring of the patient. Therefore, no MRI should be performed in sedated patients wearing a VeriChip™ device. Ethical issues, such as security of confidential patient data, increased surveillance of staff activities or concerns about the safety of implantable RFID tags were raised recently [7-12]. Today, therefore, the most promising systems of RFID tags for patient identification in hospitals are tags that are integrated into a wristband [13,14]. To ensure patient identification throughout the hospital stay it is unwanted to remove such an RFID wristband at any time.

During a hospital stay, many patients mounted with an RFID wristband will need examination by magnetic resonance imaging (MRI) or computed tomography (CT) scan. The electromagnetic fields of an MRI scanner can damage electronic devices and CT scans can influence radio transmitters by electromagnetic radiation.

Therefore, both the chip and antenna of an RFID device are potentially at risk under MRI and CT examination since generally all transponders can be destroyed by certain field forces [15]. It is not clear whether a patient wearing an RFID device under such conditions might be at risk by heat and whether relevant impairment of MRI signals occurs when RFID tags are in the MRI device. Potential risks of MRI on the RFID are beside the risk of damaging the RFID, magnetic force on potential ferromagnetic parts in tag and RF heating at the edges of the conducting materials of the RFID tag. From CT no risks for the patient are expected, but radiation could potentially damage the electronics of the Tag. To our knowledge, only one study has investigated the influence of MRI on implanted RFID tags [8], whereas no information about the safety of patients equipped with RFID wristbands under MRI and CT scan is available.

Different physical mechanisms could have an adverse effect on the RFID tags reliability. In case of MRI induced high voltages from the gradient field but also from the RF field may damage the RFID tag. In case of CT, the x-ray may influence the semiconductor structure of the RFID.

The acquired images may be influenced by the RFID. In case of MRI, slightly different magnetic properties of the RFID tag may lead to susceptibility artifacts, which could be seen as signal void or signal distortion next to the tag. In CT images, the RFID will absorb radiation, if passing the chip, leading to distorted images.

The aim of this study was to determine the reliability and data integrity of a certain sort of RFID devices under the specific conditions during clinical MRI and CT scans as well as the safety of patients wearing an RFID wristband. We hypothesize that RFIDs remain unaffected by MRI or CT examination and that the impairment of the imaging quality is not relevant in clinical routine examination.

## Methods

### Materials

**Figure 1:**
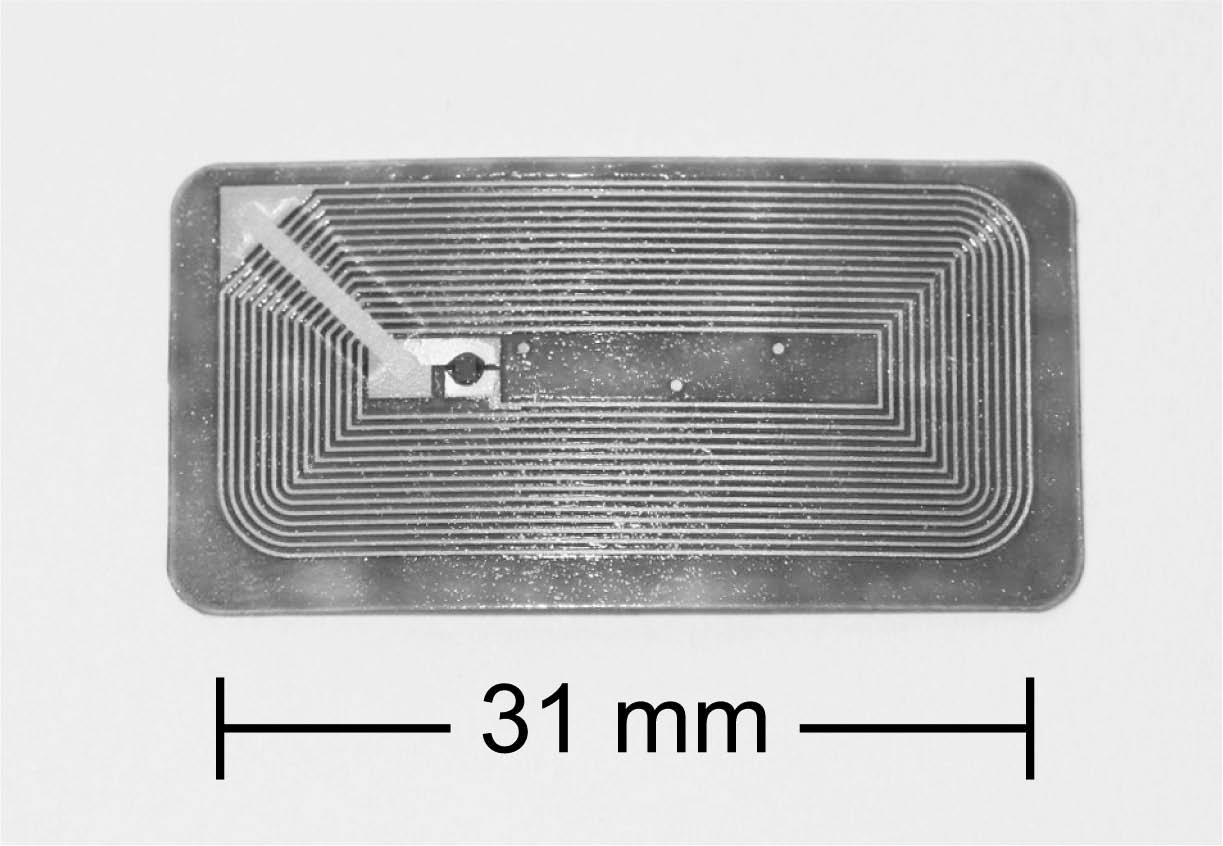
**RFID tag (31 × 14 mm, Copper, etched)**.

Sixty RFID transponder tags were provided by InfoMedis AG, Alpnach, Switzerland (equipment support). For the test, passive RFID transponder tags of the type I-Code SLI, 13.56 MHz, ISO 18000-3.1 (NXP Semiconductors, Graz, Austria) of two different sizes were used (Figure 1). These specific RFID tags were chosen because they are commonly used in high volume applications e.g. libraries, person ID or industrial applications. Further, a standardized air interface is given and used worldwide (ISO 18000-3.1; ISO 15693), the tags provide sufficient memory (> 1 k bit) and they are a low cost solution with chip prices around USD 0.10 today. The frequency at 13.56 MHz and the according reading field are suitable for required near field communication (Table 1).

**Table 1:** RFID tags used in the study

RFID Tag	Large I-Code SLI, 13,56 MHz	Small I-Code SLI, 13,56 MHz
**Chip**	1 kbit variable memory, 96 bit UID	1 kbit variable memory, 96 bit UID
**Antenna**	Dimension of antenna: 76 × 45 mm, Aluminium, etched	Dimension of antenna: 31 × 14 mm, Copper, etched

UID: Unique Identification Number

Used transponders: I-Code SLI (NXP Semiconductors, Graz, Austria)

### Methods

#### Device function

In order to perform a reliability test with the RFID transponders, 15 of each of the two types underwent MRI and CT examination. In groups of five, the RFID tags were attached to the sides of a 300 × 215 × 80 mm cardboard box in each of the three dimensions of the room. A standardized scanning protocol was used during MRI and CT scanning under the same conditions as during a patient examination. The test was performed twice, therefore a total of 60 tests of RFID tags were performed. The RIFD tags were tested for function outside the MRI room after they were scanned by the MRI machine because the reader devices contain ferrous metal.

For this clinical MRI test, a 1.5 T MR system (Avanto; Siemens Medical Solutions, Erlangen, Germany) was used and a standard protocol for a cranial examination was performed. The imaging protocol included transverse T1-weighted gradient-echo (225/4.67; flip angle, 80°) [repetition time msec/echo time msec], fluid-attenuated turbo inversion-recovery (FLAIR) (8500/111/2500 [repetition time msec/echo time/inversion time msec], T2-weighted turbo spin-echo (4000/80), diffusion-weighted (4000/102 with b-values of 0, 500, and 1000 sec/mm") and coronal fat suppressed T2-weighted turbo inversion-recovery (6000/38/140) sequences. Field of view was 210 cm; slice thickness was 5 mm with an intersection gap of 1.5 mm. Pixel size varied between 0.4 × 0.4 mm (T1-weighted images) and 0.8 × 0.8 mm (FLAIR).

Additionally, in a 3 T MR scanner (Achieva, Philips Healthcare, Best, The Netherlands) worst case sequences (high gradient performance, high B1 field and high specific absorption rate (SAR)) provided by the MR manufacturer for third party device testing have been performed continuously over nearly two hours. Four RFID tags have been placed to the edges of the field of view (FOV) where high gradient effects are expected, and four tags have been placed in the transversal plane through the isocenter (2 in the isocenter, 2 near the wall of the scanner bore). Tags have been placed parallel to the transversal, coronal and sagital imaging planes.

These worst case MR tests have been performed in a 3 T MR system (Achieva, Philips Healthcare, Best, The Netherlands). The compatibility protocols as required in the IEC 60601-2-33 [19] to evaluate influences from the MR equipment on peripheral equipment were used. On the used scanner the following three sequences have been provided which have been repeated in all three orientations. The used scanner limits the whole body SAR to 0.9 W/kg to stay within the limit of 10 W/kg for the local SAR. In the research mode this limit has been disabled and 4 W/kg could be applied. The sequence called "Max B1+SAR" was a 20 min long turbo spin-echo (131/6.1) sequence with a whole body SAR of 4 W/kg and a peripheral nerve stimulation (PNS) level of 100%, "MAX Grad" was a 3:30 min long steady-state-free-precession (2.6/0.99) sequence with a whole body SAR of 1.6 W/kg and a PNS level of 100%, and "Max Grad +RF" was a 6:30 min long turbo spin-echo (379/6.2) sequence with a whole body SAR of 3.9 W/kg and a PNS level of 100%.

In the CT scan, the exposure time was 44.6 s and a standard protocol for abdominal CT examination was performed with a CT technique of 88 mAs at 120 kV, and a Computed Tomography Dose Index (CTDI) at 6.33 rad.

An operational check was carried out on all RFID tags with a Feig Obid-MR100 RFID reader (Feig Electronic GmbH, Weilburg, Germany) before and after the imaging tests performed. Proper function of the RFID tag was defined by three criteria tested in three steps after exposure to the MR and CT scan. Firstly, the Unique Identification Number (UID) of each RFID tag must be readable without error. Secondly, correct data writing was tested by error-free programmability of the total memory area of the RFID tag. Thirdly, impeccable function of the RFID tag was assumed when the UID and the content of the variable memory of each RFID tag was again accurately tested for proper data reading.

**Figure 2:**
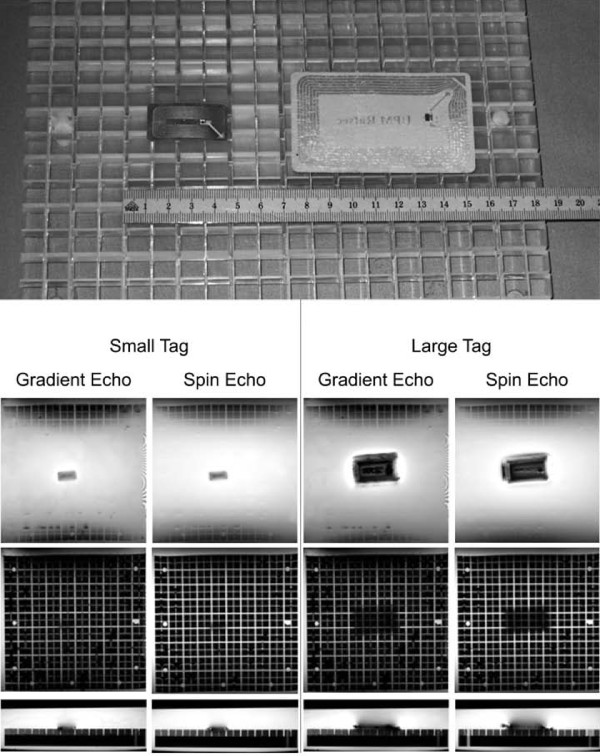
**Quantitative image artifacts**. Both RFID tags shown on the top have been placed one after the other in the middle of the plastic grid, immersed in 1 g/L CuSO4 solution. Gradient echo and Spin echo sequences as described in the ASTM Standard F2119-01 have been performed. The upper row shows MR images of a 3 mm-thick slice through the tags, the middle row slices 5 mm below the tag and the last row shows an orthogonal slice through the tag. Signal void could be found only a few millimeters away from the tag. Reduced signal due to shielding of the RF field may produce darker shadows in slices up to 1 cm away.

Artifact Assessment Signal loss has been assessed in-vitro (according to the ASTM F2119-07 Standard) and with in-vivo MRI measurements [16]. Both RFID tags have been placed one after the other in the middle of the plastic grid, immersed in 1 g/L CuSO4 solution (Figure 2). Gradient echo (TR = 100 ms, TE = 15 ms, flip angle = 30°, BW = 32 kHz) and Spin echo sequences (TR = 500 ms, TE = 20 ms, BW = 32 kHz) as described in the ASTM Standard F2119-01 have been performed in a 3 T whole body Philips Achieva MR system (Philips Healthcare, Best, The Netherlands). The resolution was 1.4 × 1.4 × 3 mm^3 ^using a 256 × 256 scan matrix.

**Figure 3:**
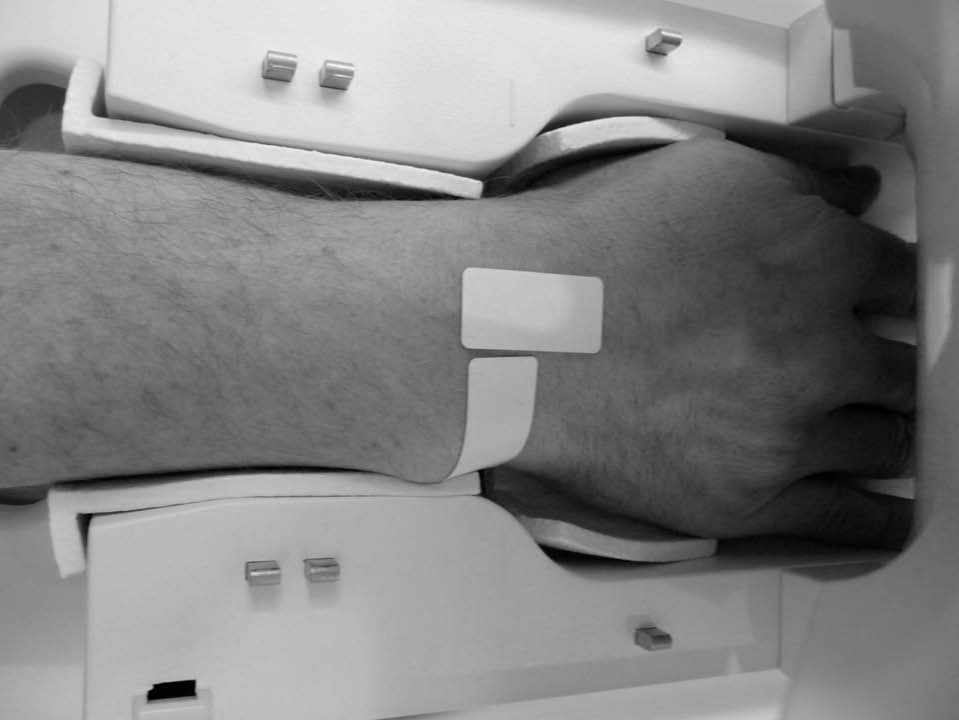
**Two RFIDs attached directly to the skin of a right wrist in a volunteer with the devices centered in a dedicated 8-channel wrist coil**.

Five of the small RFID tags were used for in-vivo tests concerning signal loss. To qualitatively measure the signal loss, a comparison of images of a wrist with and without RFID tag in the 1.5 T MR-Scanner (Avanto; Siemens Medical Solutions, Erlangen, Germany) was performed. In terms of getting a worst case scenario, an MRI of the wrist of a consenting volunteer was conducted with an RFID tag attached directly to the skin (Figure 3). The images were compared to examination results performed without an RFID device. Images were analyzed qualitatively for signal loss.

#### Patient safety

All measurements for tissue heating near the RFID tag, movement and artifacts were carried out on a 1.5 T and 3 T Philips Achieva MR system (Philips Healthcare, Best, The Netherlands) running software release 2.5.1. For the heating measurement the build in body coil was used.

### Tissue heating near RFID device

Temperature elevation is based on energy absorption. The capacity of the chip area to diffuse excessive energy generated by the antenna of an RFID tag is limited and therefore RIFD devices can heat up in higher fields [15]. However, in case of the RF field of a MRI, heating will also occur due to field distortions and elevations at or close to the metallic parts of the RFID tag. For heating measurements, the American Society for Testing and Materials (ASTM)-phantom (Standard F2182-02a) was used, which was filled with 30 l of 0.45% saline solution [17]. Measuring time was 15 minutes, and as patient weight 75 kg was entered. Since the RFID tags are not implanted but preferable wrapped around the wrist, the RFID tags were not placed inside the liquid as recommended for implants but placed on the surface of an agar-phantom mimicking the arm of a patient. The agar was made out of the same saline solution with additional 2% agar as a gelling agent. The similar setup has been used and described in detail in other contexts [20]. The agar phantom prevents convection next to the RFID. The agar-phantom was placed at the right side of the ASTM-phantom near the scanner bore wall where the most intense heating is expected. However, this is highly dependent of the position of the tag with respect to the rungs of the body resonator (high E-fields), and thus manufacturer-dependent. Due to asymmetry of the induced E-fields in the ASTM phantom the side with higher heating has to be determined with test measurements. It has been verified that the right side shows more heating effects than the left side. The RFID were placed 16 cm to the right side of the tank. A large type RFID tag was placed on the moistened agar-phantom and two temperature sensors were placed under the antenna. The entire RFID tag made contact with the agar gel in order to simulate the worst-case scenario. The reference sensor was placed directly on the surface of the agar-phantom and covered with a thick piece of paper, simulating an RFID tag with no metallic wires/content. The comparison with a placebo RFID in the same measurement allowed avoiding the known inaccuracy of the SAR estimation of clinical MR systems [18]. A further sensor was placed approximately 1 cm under the RFID tag in the agar-phantom. The tank with the RFID was moved into the scanner until; the RFID was in z-direction in the isocenter of the body coil. Measurements were performed with a specific sequence provided by the MR manufacturer according to the requested high SAR/B1 sequence in the MR standard IEC 60601-2-33 [19]. This is a Turbo Spin Echo Sequence (TSE) with a whole-body specific absorption rate (SAR) at 4 W/kg (1.5 T) and 0.9 W/kg (3 T). The 3 T MR system is limited to this whole-body SAR due to a local SAR limit of 10 W/kg. The whole body SAR provided by the scanner software was verified for the given tank with a calorimetric measurement and the the measured values were within 10% of the calculated values for both field strength. However whole body and local SAR may strongly vary for different MR systems.

### Device movement

Measurements for device movement were performed with the large and small RFID tags using the 3 T MR system (Achieva, Philips Healthcare, Best, The Netherlands) according to ASTM F2052. This is a test method that covers the measurement of the magnetically induced displacement force produced by the static magnetic field on medical devices. The main magnetic field gradient of the MR scanner used at the measurement point was 45 ± 2 mT/cm (= 4500 mT/m) [20]. It is located 3 cm away from the laser cross for patient positioning. Torque effects were assessed qualitatively and if any torque could be noticed measurements would be performed.

## Results

### Device function

All of the 60 RFID tags tested showed normal function after testing with MR and CT scans. All UID could be read before and after the test with no evidence of loss of function. Previously saved data could be read correctly and completely and the memory areas of all tags were overwritten and read properly. No loss of function or malfunction was detected. No difference between the RFID tags tested with MR or CT scan or between the two different sizes of antennas was found.

### Signal loss

**Figure 4:**
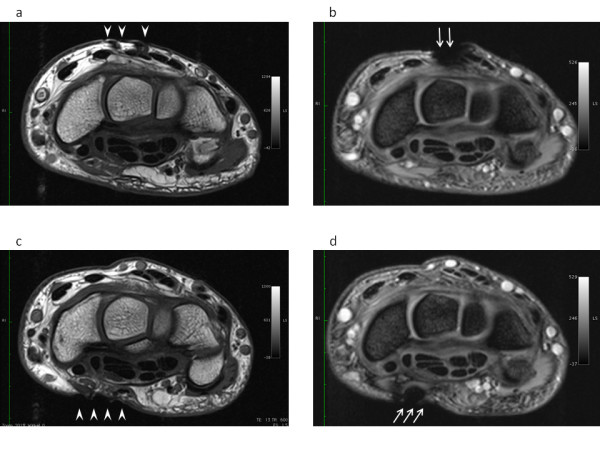
**Qualitative image artifacts**. MR imaging findings in a volunteer with the RFID tags positioned on the dorsum of the wrist (a, b) and on the volar aspect of the wrist (c, d). Axial T1-weighted spin-echo MR-image (600/13; number of signals acquired, 1; field of view, 90 mm) shows only minimal geometric distorsion and susceptibility artifacts on skin and underlying subcutaneous tissue (arrowheads) (a and c). Axial T2-weighted Flash2D gradient-echo MR-image (400/15; number of signals acquired, 2; field of view, 90 mm) shows increased susceptibility artifacts on skin and underlying subcutaneous tissue and tendons (arrows) (b and d). Interpretation of articular structures is not compromised.

Quantitative measurement of MRI signal loss showed small artifacts of about 2 - 4 mm (Figure 2). The quality of the images was minimally impaired due to the tags positioned near the body area examined (Figure 4).

No artifacts were detected in CT images and no influence of the RFID tags on the image quality was found.

### Patient safety

#### Tissue heating near RFID tag

**Figure 5:**
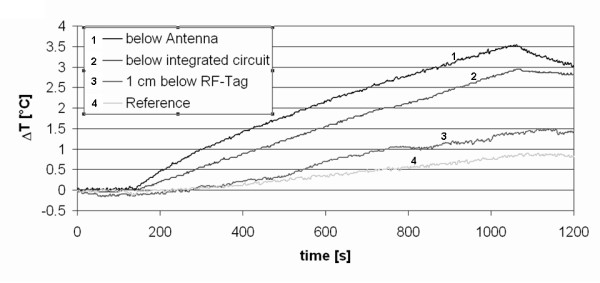
**Tissue heating near RFID tag, temperature development over time**. Temperature increases next to the large RFID tag at different positions during an MR sequence in a 1.5 T whole-body MR scanner. The temperature increases have been normalized to 2 W/kg as requested by the ASTM Standard.

With the 1.5 T MR system, normalized at 2 W/kg as required by the International Electreotechnical Commission (IEC) standard, and using the large tag, a maximum heating of 3.6°C and 3.0°C at the two different points under the tag was found after 15 minute of scanning. At the reference point on the surface of the agar-phantom, a heating of up to 1.0°C was measured. The RF power sent to the body coil was 340+-10 W. At the sensor centered 1 cm below the RFID tag, a maximum heating of 1.5°C was found. The same tests were performed with an RFID wristband containing the small RFID tag and with direct contact of the tag with the temperature sensor. With the small RFID tag, no temperature rise of more than 1.5°C was found in any tested configuration. Figure 5 shows the development of the normalized temperature over time for the worst-case measurement with the large RFID tag.

With the 3 T system all heating measurements were below 0.8°C (normalized to 2 W/kg). At 3 T, the reference sensors sometimes showed even more heating than the sensor next to the RFID tag. The RF power was in this scanner 90 ± 10 W, since the whole body SAR was limited to 0.9 W/kg.

#### Device movement

Accelerations impinging on the RFID tags were all found to be below 1 N/kg in a magnetic field gradient of 45 mT/cm, which is far below the limit of 9.8 N/kg mentioned in the standard ASTM F2052-06e [21]. Such accelerations are generally not or minimally perceptible for human beings when an RFID wristband is used. Even an RFID tag lying on a water surface was minimally moved in the main magnetic field of the 3 T system. No torque effects could be found for both tested tags.

## Discussion

This study was performed to evaluate patient safety as well as the reliability and data integrity of passive RFID devices under clinical conditions with MRI and CT scan. Passive RFID devices are likely to be used for applications with RFID wristbands for patient identification. Such patient identification is central to many other RFID-related processes in hospitals.

For the first time, we were able to show that the RFID tags used in this study sustained no damage after being exposed to typical everyday conditions during CT or MR examination. Reading and saving data was unaltered after clinical MRI or CT scans. Moreover, patient safety was not impaired by wearing an RFID wristband during MRI or CT examinations.

Within the last few years, the use of autoidentification technologies has rapidly entered the hospital environment [21]. A number of RFID applications have been implemented in hospitals so far, mostly for logistic purposes such as material tracking or inventory management, according to the original applications of the technology [2,21-23]. Only recently, more complex applications have been implemented in hospitals, for example in patient-care management processes such as blood transfusion or the prevention of wrong side surgery [1,3,13,24-27]. RFID technology seems ideal for complex environments like hospitals since the technology itself is to be applied for more gainful and differentiated applications. In this context, it was shown that autoidentification systems, ideally implemented as RFID applications, can contribute to improving efficiency and patient safety [28-30].

To ensure the proper functioning of passive RFID devices, it must be shown that no memory alterations occur and thus the ability to operate after exposure to electromagnetic fields is crucial. For the use of RFID tags in MRI systems, it is a precondition that they must not contain any ferromagnetic material. Our tags contained etched aluminum and copper, respectively, which we consider to be the important factor in finding no device movements in our tests.

All transponders had been scanned and re-written successfully each time. The large version of the tags has a 50% longer range to be read and can be used for instance as personal badges, for equipment or documents. They were tested due to their larger antenna area and consequently greater potential for energy absorption than smaller-sized RFID tags. This means that two parameters were altered in this test. The size of the antenna as well as the metal (copper in the small version and aluminum in the large version) of the RFID tags. However, no RFID tag failed in the tests.

Ferromagnetic metals are used in low-frequency RFID tags (<148.5 kHz), which are not likely to be used for the purposes described here. We used RFID tags working at 13.56 MHz because these tags are considered the most appropriate for use in a hospital environment. Today, 1.5 T MR systems operating with a frequency of 64 MHz are standard; however, low-field systems with 0.5 T or even 0.3 T operate at a frequency near that used in the RFID system tested (13.56 MHz). For these low-field systems, interference is very likely but was not tested in this study! The results presented are limited to 1.5 T and 3 T MR scanners.

The quantitative measurement showed only very small artifacts of 2 - 4 mm in size. These are minor artifacts compared to the artifacts caused by implants and do not lead to impairment of the image quality. However, we recommend not placing an RFID tag directly on the region of interest in MRI examinations (e.g. skin lesion, malignant melanoma).

According to the ASTM standard, a device is considered as MR safe if it causes no known hazards to patients in all MR environments. Since the RFID tag contains conducting materials, RFID may only be MR conditional, meaning be safe under certain conditions for MR imaging during the scan. Significant increasing of temperature and unexpected strong movements are potential risk factors for patients with an implant during MRI examination. As for the measured heating of the RFID device during MRI examination, a maximum rise in temperature of about 4°C on the skin surface is not harmful, in the normal operating mode of the MR scanner. In clinical examinations, the rise in temperature would even be reduced by the cooling effect due to sweating, air convection, blood circulation and perfusion [31].

Furthermore, our results regarding device movement showed that such concerns are irrelevant with less than 1 N/kg for MRI examinations in MRI scanners with field strength of up to 3 T in patients wearing an RFID wristband. No torque effects could be seen.

Although the types of RFID tags tested in our study are those most likely to be used in hospitals, we are aware of the fact that there is a huge market of different types of RFID tags. These other types of RFID tags need to be tested in different MR conditions and also under other clinical conditions. Given that we used passive RFID tags, no statement can be made about active RFID tags under the clinical conditions described. However, the utilization of the much cheaper passive RFID tags in hospitals is much more likely than the more expensive active tags.

## Conclusion

Wristbands for patient identification equipped with RFID transponders not containing ferromagnetic components do not have to be removed for MRI and CT scanning. This ensures that patient identification is guaranteed without interruption and no data loss is expected.

We conclude that patients wearing RFID wristbands are safe in 1.5 T and 3 T MR scanners using normal operation mode for RF-field, when the types of tags tested here are used. Our conclusions pertain specifically to the RFID tags that underwent evaluation.

## Abbreviations

ASTM: American Society for Testing and Materials; CT: Computed tomography; CTDI: Computed tomography dose index; FLAIR: Fluid-attenuated turbo inversion-recovery; FOV: Field of view; HF: High frequency; LF: Low frequency; MRI: Magnetic resonance imaging; PNS: Peripheral nerve stimulation; RFID: Radio frequency identification; SAR: Specific absorption rate; TSE: Turbo Spin Echo Sequence; UHF: Ultra-high frequency; UID: Unique identification number

## Competing interests

One of the authors (CK) is Chief Technical Officer at InfoMedis AG, Alpnach, Switzerland

## Authors' contributions

TS developed the idea of the study, participated in the study design and drafted the manuscript. RL performed the experiments at ETHZ laboratory and personally approved the correct appliance of the here used methodological standards with the competent authorities from FDA. SW and CF performed the clinical experiments at KSSG and drafted the manuscript. CK participated in the study design and contributed specific RFID know-how. JL and FHH participated relevantly in drafting the manuscript. All authors read and approved the final manuscript.
